# Identifying preeclampsia-associated genes using a control theory method

**DOI:** 10.1093/bfgp/elac006

**Published:** 2022-04-28

**Authors:** Xiaomei Li, Lin Liu, Clare Whitehead, Jiuyong Li, Benjamin Thierry, Thuc D Le, Marnie Winter

**Affiliations:** UniSA STEM, University of South Australia, Mawson Lakes, 5095, SA, Australia; UniSA STEM, University of South Australia, Mawson Lakes, 5095, SA, Australia; Pregnancy Research Centre, Dept of Obstetrics & Gynaecology, University of Melbourne, Royal Women’s Hospital, Melbourne, 3052, VIC, Australia; UniSA STEM, University of South Australia, Mawson Lakes, 5095, SA, Australia; Future Industries Institute, University of South Australia, Mawson Lakes, 5095, SA, Australia; UniSA STEM, University of South Australia, Mawson Lakes, 5095, SA, Australia; Future Industries Institute, University of South Australia, Mawson Lakes, 5095, SA, Australia

**Keywords:** preeclampsia, gene, association, control theory method

## Abstract

Preeclampsia is a pregnancy-specific disease that can have serious effects on the health of both mothers and their offspring. Predicting which women will develop preeclampsia in early pregnancy with high accuracy will allow for improved management. The clinical symptoms of preeclampsia are well recognized, however, the precise molecular mechanisms leading to the disorder are poorly understood. This is compounded by the heterogeneous nature of preeclampsia onset, timing and severity. Indeed a multitude of poorly defined causes including genetic components implicates etiologic factors, such as immune maladaptation, placental ischemia and increased oxidative stress. Large datasets generated by microarray and next-generation sequencing have enabled the comprehensive study of preeclampsia at the molecular level. However, computational approaches to simultaneously analyze the preeclampsia transcriptomic and network data and identify clinically relevant information are currently limited. In this paper, we proposed a control theory method to identify potential preeclampsia-associated genes based on both transcriptomic and network data. First, we built a preeclampsia gene regulatory network and analyzed its controllability. We then defined two types of critical preeclampsia-associated genes that play important roles in the constructed preeclampsia-specific network. Benchmarking against differential expression, betweenness centrality and hub analysis we demonstrated that the proposed method may offer novel insights compared with other standard approaches. Next, we investigated subtype specific genes for early and late onset preeclampsia. This control theory approach could contribute to a further understanding of the molecular mechanisms contributing to preeclampsia.

## 1 Introduction

Preeclampsia (PE) is a hypertensive disorder of pregnancy and a leading cause of maternal and neonatal mortality and morbidity globally. Clinically it is associated with high blood pressure after 20 weeks of gestation, and proteinuria or end organ damage [1]. PE affects 5–8% of pregnancies, resulting in complications for both the mother and her offspring, including fetal growth restriction (FGR), organ damage (liver/kidney/brain) and even fetal and/or maternal death [2].

Currently, the only definitive treatment for PE is delivery of the placenta (and the baby) and while originally thought to resolve soon after birth, the disease is now known to have long-term ramifications for mothers and their offspring, including an increased risk of cardiovascular disease and diabetes later in life [2]. However, early detection of pathological changes or identification of ‘high-risk’ pregnancies could enable early intervention and management. For example, low dose aspirin given to women at high risk before 16 weeks of gestation reduces the risk of PE development [3]. Many other treatments have been investigated although these often treat the symptoms of the condition rather than the cause and are aimed at prolonging pregnancy [4]. One reason for this is that while the clinical symptoms of PE are well defined, its precise molecular pathogenesis and origins are not well understood [5]. This is compounded by the fact that the heterogeneous clinical presentation of PE generally manifests later in gestation, while the molecular triggers occur much earlier in pregnancy.

PE is a multifactorial condition that varies widely in severity and is the result of complex interactions between maternal and fetal genotypes, as well as environmental factors [6]. This, ultimately, results in structural placental damage and ischemia, the release of anti-angiogenic and pro-inflammatory cytokines into the maternal circulation resulting in endothelial dysfunction which leads to the clinical manifestations.

To account for the multifactorial origin of PE, mathematical and computational models have been used to evaluate the pathophysiology as well as to identify or test biomarkers predicting or diagnosing the syndrome. The combination of maternal characteristics, medical history, ultrasound and first trimester maternal serum biomarkers (PlGF and PAPPA) can predict 90% of pregnancies at high risk for early onset PE (EOPE) development (10% false discovery rate) [7]. As such this approach of combining clinical and biochemical markers has been recommended by the International Federation of Obstetrics and Gynecologists [8]. This advance in combining biomarkers with clinical parameters is clinically highly relevant, as the risk of EOPE can be mitigated if aspirin is commenced early in these pregnancies [9]. However, these biomarkers perform poorly for late onset PE (LOPE), identifying only 40% and therefore there is a great need to improve prediction of risk in these pregnancies.

In the second and third trimester biomarkers hold great promise to improve prediction of PE and identify those potentially at highest risk of adverse maternal or fetal outcomes [10]. The ratio of sFlt-1 and PIGF can be used to predict PE development within 4 weeks in women with the suspected syndrome (24–36 weeks of gestation) [11] and is validated clinically to rule out the condition in symptomatic pregnancies [12]. However, this current approach has limited clinical utility as although they are able to obtain a high negative predictive value (ability to identify women who will not develop PE), they often suffer from poor positive predictive values and cannot be used to ‘rule-in’ disease. Ultimately, the identification and implementation of biomarkers or disease signatures with a high positive predictive value would be hugely significant. Improved understanding of the molecular mechanisms which lead to PE including the identification of critical biomarkers would improve clinical outcomes for the mother and offspring, through improved diagnosis, treatment and management.

Due to the great clinical need, a wide array of research approaches utilizing various types of genomic data and/or transcriptomic data have been used to identify PE-associated biomarkers and understand disease mechanisms [13]. Most prior research has adopted differential expression (DE) analysis to identify PE-associated biomarkers. For example, DE analysis has been applied to PE gene expression data [14, 15], miRNA expression data [16], lncRNA expression data [17] and DNA methylation data [18] to identify the genetic susceptibility of PE. In DE analysis, genes, miRNAs, lncRNAs or DNA methylations are tested individually for expression differences between PE and control groups. The differentially expressed genes (DEGs) are important for understanding the link between genotypic and phenotypic variation [19].

To elucidate PE molecular mechanisms, some research has established and analyzed interaction networks. Network analysis involves building a biological network based on a curated gene set from DEGs [20], the literature [13, 21] or other biological information [22]. Then, network-based methods are adopted to identify critical PE-associated genes from the gene list [21]. For example, based on the protein–protein interaction (PPI) network for a set of 347 genes, Tejera *et al.* demonstrated that the five genes with the highest hub scores were well-known protein markers of PE [21]. This hub analysis has also been used in several works for identifying PE biomarkers [23, 24]. A more recent example used Google PageRank algorithm to determine important placental genes from the protein interaction network identifying genes which may have relevance in placenta-related disease but which are not necessarily placental specific [25].

Both DE analysis and some network analysis approaches have their limitations such as the ability to only use one type of data (such as transcriptomic or network). For example, DE analysis only tests the relationship of PE and genes based on the hypothesis that genes are independent [26], however, genes can significantly interact. Network analyses can leverage gene regulatory relationships in identifying PE-associated genes, but most current works map the gene sets to a general PPI network [21, 25] which is not specific to PE. Thus, the PPI network might include some interactions that do not exist in PE.

In order to address the limitations of current methods, we propose a method (termed cPE) that utilizes both transcriptomic and network data. In this method, we firstly construct a network for a condition (e.g. PE) from the gene expression data. We then orientate this network with the directed PPI network and filter out the links that do not exist in the PPI. Thus, we eliminate interactions that do not exist in PE and the resulting network is more reliable than that solely derived from gene expression data. Then, we apply controllability analysis to identify critical PE-associated genes from the developed PE-specific network.

Controllability analysis [27] provides an understanding of the PE-specific network and the different roles that nodes play in the network. In control theory, a system is controllable if it can be driven from any state to any expected state within a finite time by suitable inputs. Based on this theory, Liu *et al.* developed an analytical toolbox to study the controllability of a directed network [27]. This toolbox has been successfully applied to identify cancer genes [28] and breast cancer drivers [29]. It is well known that there are many parallels between cancer and pregnancy in growth, invasion and immune mechanisms [30] and this controllability method developed for cancer will likely have utility in pregnancy and in particular PE. Herein, controllability analysis was used to capture two types of critical nodes from the developed PE specific network. Both types of critical nodes play important roles in the controllability of the gene regulatory network and we considered the critical nodes as important in PE disease mechanisms. Finally, the critical PE-associated genes were prioritized using the log fold-change (LogFC) data from the DE analysis. We demonstrated that the proposed method yields a higher proportion of validated PE-associated genes than current methods, DE analysis, betweenness centrality and hub analysis for the specified dataset. The predicted critical PE-associated genes are also supported by a PE-related SNPs database, a literature-based database and known protein markers for PE. Besides the recovery of known PE-associated genes, cPE identified genes with little known PE-associations, which are enriched in the same GO terms and KEGG pathways as validated PE-associated genes, suggesting their important roles in PE development and the effectiveness of our approach.

## 2 Materials and methods

### 2.1 Dataset

We applied cPE to a microarray dataset that contained both PE and control samples to identify PE-associated genes. The gene expression data and clinical data were downloaded from the Gene Expression Omnibus database^1^ under the accession number GSE75010. The clinical data included information regarding patients’ phenotype and gestational age. The transcription factor (TF) list was obtained from the FANTOM5 transcriptome catalog database [31]. The directed PPI network was downloaded from the DirectedPPI database^2^ [32]. The directed PPI network contains the general human directed PPI network, which helped to build the PE gene regulatory network. The PE-related SNP data were downloaded from the PESNPdb database^3^ (version 1.1).

**Figure 1:**
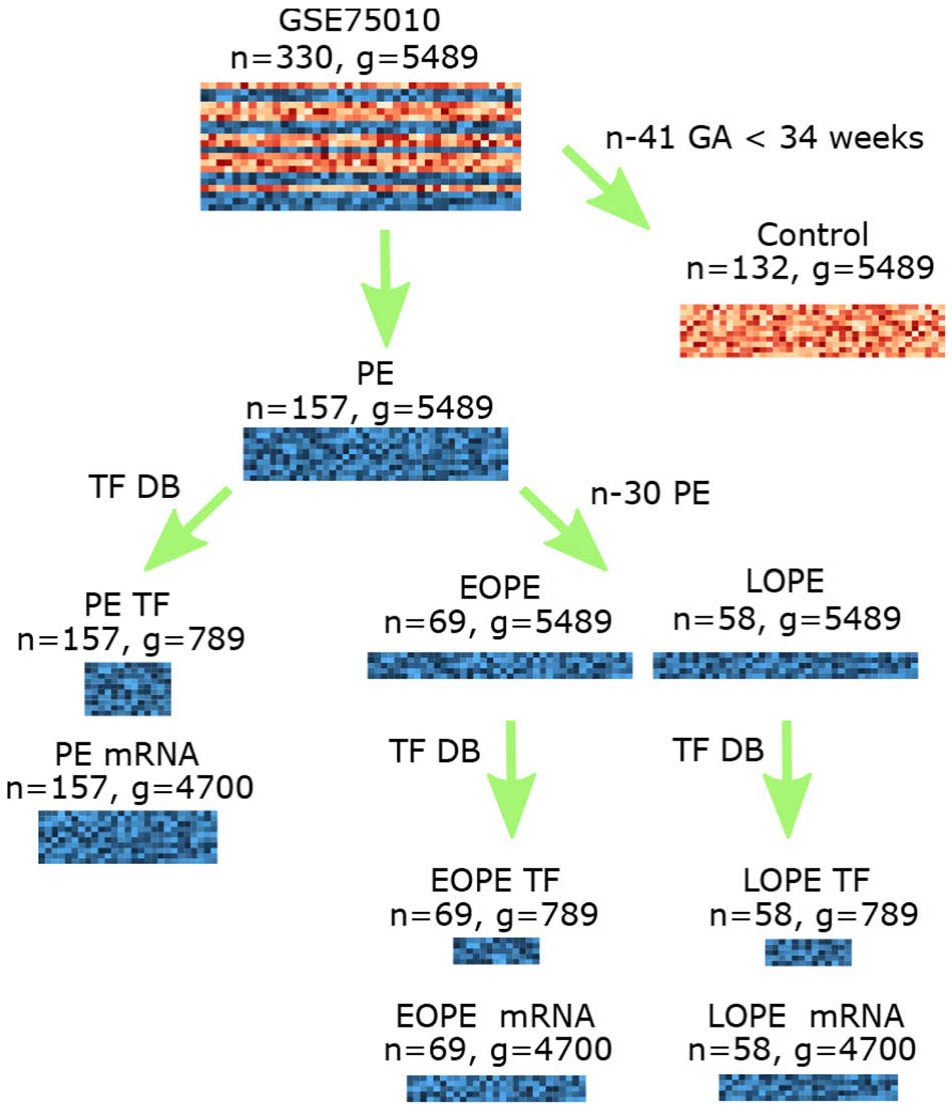
Data preprocessing. The control dataset only contains term samples whose Gestational Age (GA) is more or equal to 34 weeks. Transcription Factor (TF) coding messenger RNAs (mRNAs) and other mRNAs are classified using the TF list from a TF database (DB). n: number of samples. g: number of genes. Note: 41 samples with gestational ages less than 34 weeks (i.e. preterm control samples) were removed from GSE75010, therefore, only term control samples were used in the analysis. When stratifying preeclampsia samples into the EOPE and LOPE subtypes, 30 preeclampsia samples were removed from the dataset as associated gestational ages were unknown.

GSE75010 was chosen as it is one of the largest publicly available collated datasets of human placental microarray data, containing 157 samples from PE placentas and 173 control samples. GSE75010 was generated by integrating eight placental gene expression datasets after removing eventual biases [33]. Of note, DEG discoveries often lack reproducibility among different microarray studies due to their small sample size and the presence of systematic variation across studies. Conducting a DE analysis based on an integrative dataset yields more robust DEG results than the DE analysis on individual datasets [34]. For identifying PE-associated genes, we divided GSE75010 into two subsets (Figure 1 and S1 File): PE and control expression sub-datasets. During the process, 41 preterm control samples in GSE75010 were removed with only term controls used.

In order to detect critical genes for PE subtypes, GSE75010 was further divided into either EOPE or LOPE datasets (Figure 1) based on gestational age with a cutoff of 34 weeks. For this analysis 30 PE samples without gestational age information were removed. As a result, the EOPE dataset contained 69 samples and the LOPE dataset consisted of 58 samples.

For the expression data of each condition (PE, EOPE or LOPE), we prepared matched Transcription Factor coding messenger RNA (TF for short) and other mRNA (mRNA for short) data to construct the gene regulatory network. TFs and mRNAs were classified using the TF list from the FANTOM web resource. To integrate the transcriptomic and network data, only genes with gene expression profiles in GSE75010 and link(s) in the DirectedPPI database were selected. In total, 789 TFs and 4700 mRNAs were obtained.

### 2.2 Methodology

**Figure 2:**
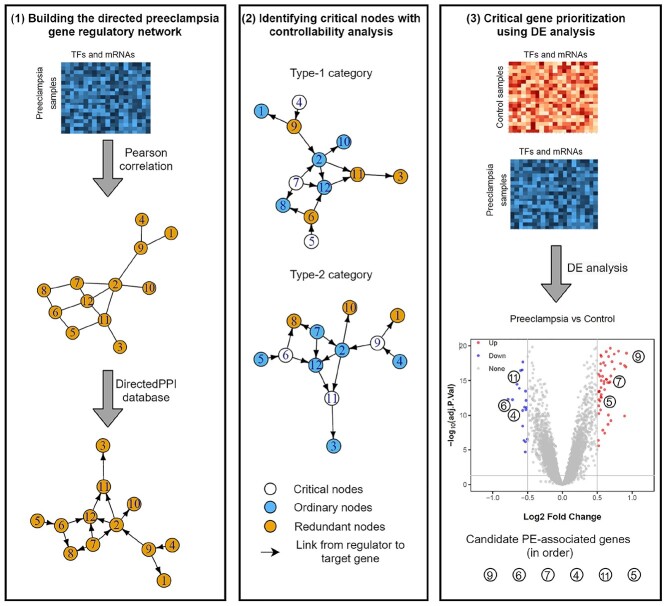
A schematic framework of the control theory approach to identify preeclampsia-related genes. (1) From the matched transcription factor (TF)/messenger RNA (mRNA) expression data from preeclampsia samples, Pearson correlation was used to construct an undirected gene–gene network. This network was then orientated based on the DirectedPPI database. The nodes of the directed preeclampsia gene regulatory network are TFs or mRNAs, and the links start from regulators to their target genes. (2) Controllability of the directed preeclampsia gene regulatory network and categorized nodes were analyzed. Two types of critical nodes were identified. (3) The expression data of preeclampsia and control samples were used for differential expression (DE) analysis. Lastly, the critical nodes were ranked by their log fold change values.

The proposed cPE method has three key steps to identify PE-associated genes (Figure 2): (1) Development of a directed PE gene regulatory network, (2) Identification of critical nodes via controllability analysis and (3) Prioritization of critical genes using DE analysis.

#### Constructing the directed PE biological network

The directed condition biological network was constructed using the matched TF and mRNA expression data for a disease condition (PE) and the directed PPI network from DirectedPPI [32]. This network contains two types of regulatory interactions, including those between TF$\rightarrow $TF/mRNA and mRNA$\rightarrow $mRNA. Although TFs are the major regulators in the gene regulatory network, knowledge about regulating TFs is incomplete, meaning some mRNAs are potentially playing the role of TFs [35]. Therefore, links between mRNA–mRNA have been included if they are strongly correlated and exist in the directed PPI network. The network was constructed as follows:

- Identify the link between nodes. Pearson correlation coefficient (PCC) was used to test for associations between all paired nodes. The significance of PCCs was evaluated by the *P*-value of the PCC test. To reduce the false discovery rate of identified gene links, only statistically significant links whose Benjamini–Hochberg adjusted *P*-values are less than 0.05 were retained [36].
- Orientate the obtained links. We orientated the obtained links in the network as follows. First, we oriented the links based on the following assumption: TFs can regulate TFs and mRNAs, and mRNAs can regulate mRNAs [29]. Second, we refined the network by removing the links which are not in the directed PPI network. We built a reliable directed condition-specific gene regulatory network using the prior network information.

In the directed biological condition network, the number of links toward a node is the in-degree and the number of links away from the node is the out-degree. The sum of in-degree and out-degree of a node is its degree.

#### Identifying critical nodes via controllability analysis

Given a linear dynamic system (network) with $N$ nodes $x_{1},x_{2},\ldots ,x_{N}$, an adjacency matrix $A_{N\times N}$ holds the interaction strength between nodes. The system can be defined by a set of linear ordinary differential equations:

(1) \begin{align*} \frac{d\mathbf{x}(t)}{dt}=A\mathbf{x}(t)+B\mathbf{u}(t), \nonumber\\ \mathbf{x}=(x_{1}(t),x_{2}(t),\ldots,x_{N}(t))^{T}, \end{align*}

where $x_{i}(t)$ indicates the node state at time $t$, and $B_{N \times M}$, $(N \geq M)$ is the input matrix that describes $M$ nodes controlled by an external controller. Only the diagonal elements in $B_{N\times M}$ are nonzero and they capture the interaction strengths between the controller and its target node. The $M$ corresponding nodes are called driver nodes as first described in [27]. The linear dynamic system is controlled by the input signal $\mathbf{u}(t)=(u_{1}(t),u_{2}(t),\ldots ,u_{M}(t))$ that is manipulated by the controller. Based on Kalman’s controllability rank condition [37], the linear dynamic system can be controllable if and only if the controllability matrix $C_{N\times NM}=(B,AB,A^{2}B,\ldots ,A^{N-1}B)$ has a full rank:

(2) \begin{align*} \textrm{rank}(C) = N. \end{align*}

Furthermore, if it is possible to select binary matrices $A$ and $B$ that satisfy Equation 2, a complex network is deemed to be a locally structural controllable [28].

We hypothesized that the obtained biological network is a linear dynamic system and can be controlled by an external controller. Therefore, controllability analysis can be applied to the biological network even without interaction strength information. A graph-based method developed by Liu *et al.* was used to identify all the sets of driver nodes that satisfy Kalman’s controllability rank condition [27]. Then node categories were implemented based on the identified sets of driver nodes. We defined six types of nodes, including two critical, two ordinary and two redundant from two types (termed type-1 and type-2) of node category methods.

**Figure 3:**
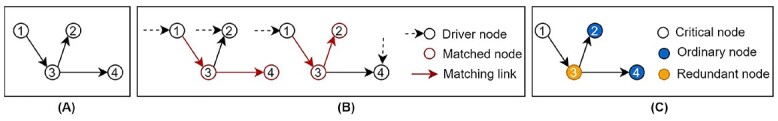
Type-1 node categories. **(A).** Input network. **(B).** Search for driver node sets. The network can be fully controlled if and only if one of the driver node sets {1, 2} or {1, 4} is driven by controllers. **(C).** Type-1 node categories. Based on the definition of type-1 node categories, node 1 is a critical node, 2 and 4 are ordinary nodes and 3 is a redundant node.

In type-1 node categories, a critical node must be in all sets of driver nodes of the network, an ordinary node is present in at least one of the sets of driver nodes and a redundant node does not appear in any of the sets of driver nodes (Figure 3). Thus, in order to control the network, all type-1 critical nodes should be controlled. In the type-1 node category method, we searched for a maximum set of links defined as the group of links that satisfy the following two conditions: (i) there are no two links in the group sharing the same start node or the same end node, (ii) the number of elements in the set is the maximum. The links in the maximum set are known as matching links. Using the matching links, the nodes were classified in the networks as matched or unmatched nodes. A node was considered a matched node if any of its links belong to the set of matching links and it was unmatched otherwise. Since the network can be fully controlled only if the unmatched nodes are controlled [27], we define the set of unmatched nodes as the set of drivers.

It is important to note that the set of matching links is not necessarily unique. To clarify this, we assume an input network shown in Figure 3A. In this example, two sets of links are identified as a set of matching links, i.e. set $\{1\rightarrow 3,3\rightarrow 4\}$ and set $\{1\rightarrow 3,3\rightarrow 2\}$ (Figure 3B). Thus, the set of drivers is also not unique. Following this example, we have two sets of drivers, i.e. set $\{1,2\}$ and set $\{1,4\}$. By definition, critical nodes must be in all sets of driver nodes. In our example, only node $1$ is considered a critical node (Figure 3C).

**Figure 4:**
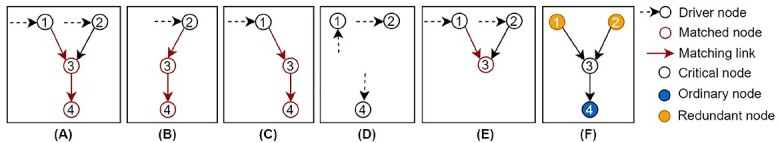
Type-2 node categories. **(A).** Input network. Size of minimum driver node set $|\textrm{MDNS}|=2$. **(B).** Removing node 1. $|\textrm{MDNS}|=1$. **(C).** Removing node 2. $|\textrm{MDNS}|=1$. **(D).** Removing node 3. $|\textrm{MDNS}|=3$. **(E).** Removing node 4. $|\textrm{MDNS}|=2$. **(F).** Type-2 node categories.

There are several sets of driver nodes that can satisfy Equation 2, but the minimum size of the driver node sets (MDNS) is unique. In type-2 node categories, a node is critical if its absence causes a rise in the size of the MDNS. The type-2 redundant nodes are those whose absence does not affect the driver node sets. The nodes that are neither critical nor redundant are called type-2 ordinary nodes. Figure 4 illustrates the effect of a node removal on MDNS size and its type-2 node category. Based on the definition of type-2 node categories, node 3 is a critical node, 1 and 2 are redundant nodes and 4 is an ordinary node.

From the definition of type-1 and type-2 nodes categories, we can see that the network could be controllable with type-1 critical nodes driven at all times and in the absence of type-2 critical nodes with more interactions on driver nodes. Therefore, type-1 and type-2 critical nodes are both considered critical PE-associated genes. These critical nodes may work together to control the whole network and respond to the external controllers to transform the biological state from a normal to a preeclamptic phenotype.

#### Prioritizing critical genes

Identified critical PE-associated genes that were likely to be involved in PE were ranked using DE analysis. Two typical outcomes of DE analysis are the level of differential expression (log fold change of expression, LogFC) and the significance of the difference (indicated by *P*-value) between disease and control conditions. LogFC and *P*-value have been widely used in ranking and selecting candidate genes for diseases, including PE [14, 15], preterm birth [38], cancer [39] and more as discussed by Rodriguez-Esteban *et al.* [40]. The bigger LogFC of a gene, the more likely the gene is involved in PE development. Additionally, if the *P*-value (adjusted by Benjamini–Hochberg method, adj.P.val) of a gene is greater than 0.05, it is not considered significantly differentially expressed between disease and control conditions, and it is not used for LogFC value ranking.

DE analysis was performed between a disease condition and its control groups based on their gene expression data. We then ranked the critical PE-associated genes by the decrease of their LogFC values (if *P*-values $\leq $ 0.05). Based on different conditions, PE-associated genes, EOPE-associated genes and LOPE-associated genes were identified and ranked.

### 2.3 Enrichment analysis

We subsequently used GO and KEGG pathway enrichment analysis to investigate the identified novel PE-associated genes and to understand underlying mechanisms by which PE affects placental physiology, since dysregulation of genes and biological pathways could contribute to abnormal behavior in PE. Specifically, we used the 17,913 human genes annotated in the org.Hs.eg.db [41] package (version 3.8.2) as the background. Then, the clusterProfiler [42] package (version 4.0) was used to implement the enrichment analysis on genes of interest against the background. We use the REVIGO tool [43] to remove the redundant GO terms of the enriched GO terms. Therefore, no two GO terms are more similar than 0.7 in the nonredundant GO term set. The semantic similarity between two GO terms is measured by a graph-based method proposed by Wang *et al.* [44]. The similarity of two GO term lists are computed by the *mgoSim* function in the GOSemSim package [45].

### 2.4 Differential expression analysis

The differential gene expression analysis between disease (e.g. PE) and control groups was performed by the Limma package [46]. We used the Benjamini–Hochberg method to control the False Discovery Rate (FDR) for multiple hypothesis testing in the Limma package. In DE analysis, the genes significantly differentially expressed in two groups (FDR < 0.05) were selected and ranked based on the LogFC values. In this study, the DE analysis was used for ranking PE-associated genes identified by cPE and as a comparison method for cPE.

### 2.5 Network analysis

Network analysis was conducted to investigate network properties, including scale-free structures and hub nodes. A network is claimed to be scale-free when the fraction of nodes with degree $k$ follows a power law as

(3) \begin{align*}& Pr(k) \sim k^{-\lambda}, \end{align*}

where $\lambda $ is a scaling exponent. The gene regulatory network is a directed network, hence in- and out-degree distribution data were fitted into two power-law models satisfying Equation 3, respectively. The values of $\lambda $ for in- and out-degree distribution were estimated by the R package poweRlaw [47]. We evaluated the goodness-of-fit of the fitted model using a bootstrap approach [48] that estimated a *P*-value. If the *P*-value is more than 0.1, we considered the network to be scale free as is the practice described in [49]. The most notable characteristic in a scale-free network is the presence of large hubs, i.e. highly connected gene nodes.

The gene regulatory network had a number of hubs that were more likely to be crucial than other nodes [50]. In this study, Kleinberg’s hub centrality scores (hub scores for short) were used to identify hub genes in a gene regulatory network. In addition, based on the gene regulatory network, betweenness centrality (BC) has been widely applied to explore candidate disease genes [51]. Genes with high betweenness centrality scores play an important role in disease since they control the information flow of the gene regulatory network [51]. The functions *hub_score()* and *betweenness()* in the igraph package [52] were used to calculate hub and BC scores, respectively. In the hub/BC analysis, genes with positive hub/BC scores were retained and ranked based on their hub/BC scores.

## 3 Results

### 3.1 Characterizing the controllability of the PE-specific network

**Figure 5:**
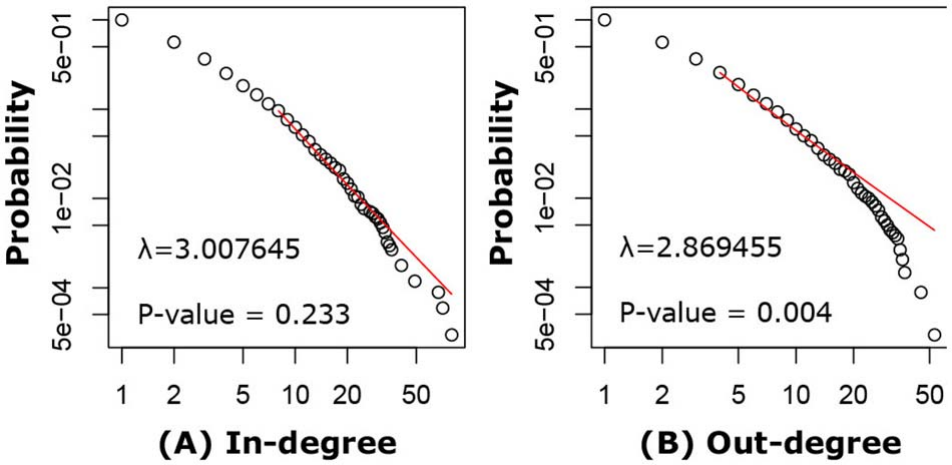
Characteristics of the PE-specific network. The PE-specific network had power-law in-degree distribution (A) but did not have power-law out-degree distribution (B). The red lines indicate the fitted power-law models.

The constructed PE-specific network consists of 5489 nodes (including 789 TFs and 4700 mRNAs) and 11,126 directed links (including 982 TF-TF links, 705 TF-mRNA links and 9439 mRNA–mRNA links). DirectedPPI contains 28,870 links among these 5489 nodes, suggesting about 38.5% of links from the general directed PPI occur in PE. For the in-degree distribution, the PE-specific network was scale free (Figure 5A). However, the PE-specific network was not a scale-free network for the out-degree distribution (Figure 5B). Taken together, the PE-specific network was not scale free. Not surprising as it has been reported that strong scale-free networks are rare in the real world [49]. In the network, the average degree is approximately 5, and there are a total of 2026 driver nodes, accounting for 45.84% of the nodes in the constructed PE-specific network.

**Figure 6:**
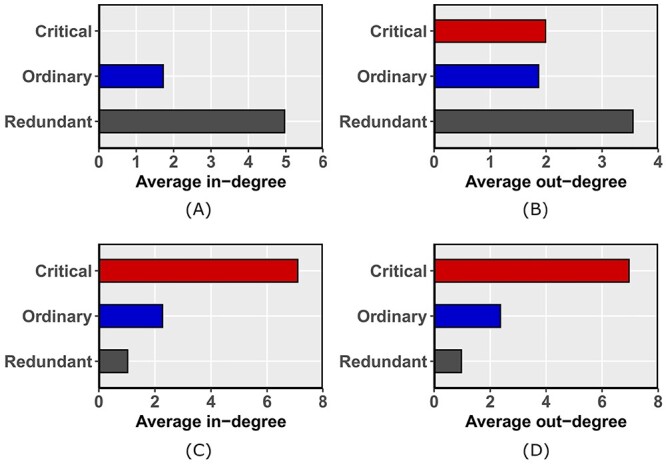
Characterizing the degrees of type-1 and type-2 nodes. Average in-degree (A) and out-degree (B) for type-1 nodes. Average in-degree (C) and out-degree (D) for type-2 nodes.

We applied controllability analysis on the network and then classified the nodes as type-1 critical, ordinary and redundant based on whether they belong to all, any or none of the driver node sets, respectively. In the network, 23.53% of nodes are critical, 39.48% are ordinary and the remaining 36.99% are redundant (Table 1). Interestingly, type-1 critical nodes had lower in- and out-degrees compared with ordinary and redundant nodes (Figure 6). Most network analysis is aimed at finding hub nodes or central nodes which tend to have high degrees, and do not identify the type-1 critical nodes in the network.

**Table 1:** Number of nodes in type-1 and type-2 categories

Node type	Type-1	Type-2
Critical nodes	1040 (23.53%)	682 (15.43%)
Ordinary nodes	1745 (39.48%)	1974 (44.66%)
Redundant nodes	1635 (36.99%)	1764 (39.91%)

The nodes were further classified in the network as type-2 critical, ordinary and redundant based on the effect of node removal on MDNS size. 15.43% of nodes are critical, 44.66% are ordinary and the remaining 39.91% are redundant (Table 1). The three types of nodes have heterogeneous degree distributions. The critical nodes have higher in- and out-degrees compared with ordinary and redundant nodes (Figure 6). These critical nodes may be more important than other nodes, since their absence isolates other nodes and is likely to disrupt the function of biological pathways.

### 3.2 cPE is effective in identifying PE-associated genes

To confirm PE gene associations identified with cPE, a literature-based relational database (dbPEC) [53] was used as a comparison and to confirm overlap with previously validated PE-associated genes. The dbPEC database was created to collect genes and gene variants associated with PE by mining published literature for potential genetic associations with PE-related phenotypes (last updated 23 October 2015). This database was used and contains both maternal (intervillous space, basal plate, myometrium, peripheral maternal blood or chorio-decidual blood) and fetal (placenta, amnion, umbilical arteries/veins, umbilical vein endothelial cells) derived genes. dbPEC contained 2781 unique genes from 1082 articles (provided by Dr Alper Uzun from Brown University via personal communication). Among these genes, 601 genes were statistically significantly associated with PE based on 899 original research articles (downloaded from https://dbpec.brown.edu/). Although dbPEC is incomplete (missing associations published after last database update and associations still to be established), it is, however, currently the most comprehensive database for validation.

**Figure 7:**
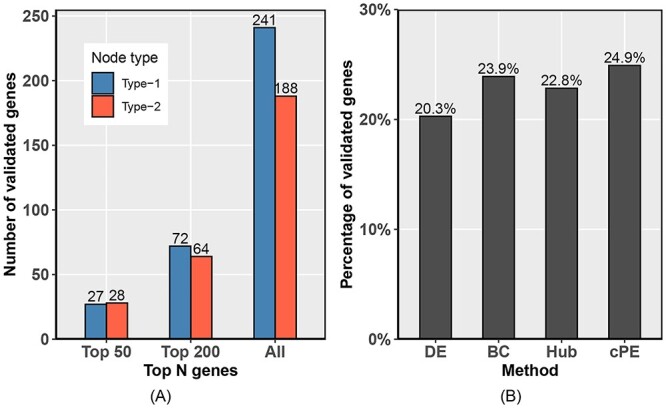
Validation using dbPEC. **(A)**. The two types of critical nodes which also appear in dbPEC. Each bar in the chart indicates the number of validated genes in one case (top 50, 200 or all critical nodes) for each type. **(B)**. The percentage of known PE-associated genes (as listed in dbPEC) recovered by cPE and three existing methods. The X-axis lists four methods differential expression (DE) analysis, betweenness centrality (BC) analysis, hub (Hub) analysis and the proposed method cPE. All the methods were applied to the same gene expression dataset or preeclampsia-specific network. The Y axis is the percentage of validated genes using dbPEC.

We found that both type-1 and type-2 critical nodes have overlap with dbPEC (Figure 7A), which indicates that cPE does identify validated PE-associated genes. We defined type-1 and type-2 critical nodes as PE-associated genes. In order to further rank for relevance, we utilized LogFC values. The bigger the LogFC of a gene, the higher it is in the ranking list. The overlap between top critical nodes (top 50, and 200) of type-1 and type-2 and dbPEC was investigated (Figure 7A). Overall, 89 type-1 and 87 type-2 critical nodes were reported to be significantly associated with PE, demonstrating that both type-1 and type-2 critical nodes have yielded distinct validated genes, which means both type-1 and type-2 critical nodes contribute in identifying PE-associated genes. It is important to note that type-1 critical nodes cannot be type-2 critical nodes and vice versa. When combining the two types of critical nodes, we obtained 1722 PE-associated genes including 429 genes validated in dbPEC.

We then compared the performance of cPE with three existing methods DE, BC and Hub analysis (Figure 7B). The DEGs of DE analysis recovered 20.2% (831/4096) of PE-associated genes and 4.2% (172/4096) significant PE-associated genes validated in dbPEC. The BC analysis recovered 23.9% (554/2317) of PE-associated genes and 9.1% (210/2317) significant PE-associated genes validated in dbPEC. The Hub analysis recovered 22.8% (996/4370) of PE-associated genes and 7.8% (342/4370) significant PE-associated genes validated in dbPEC. The critical PE-associated genes in cPE recovered the highest ratio (24.9%, 429/1722) of PE-associated genes and (10.2%, 176/1722) of significant PE-associated genes within dbPEC. Compared with the other three methods, cPE identified a smaller number of genes but a higher percentage of PE-associated genes and significant PE-associated genes which are also listed in the dbPEC database. A significant number of PE-associated genes identified by cPE are confirmed by dbPEC (the hypergeometric *P*-value = 1.96e-07). Therefore, it is statistically unlikely that the whole gene list is randomly selected and hence must be associated with PE. Importantly this can help researchers reduce the number of irrelevant candidates for wet lab validation.

**Figure 8:**
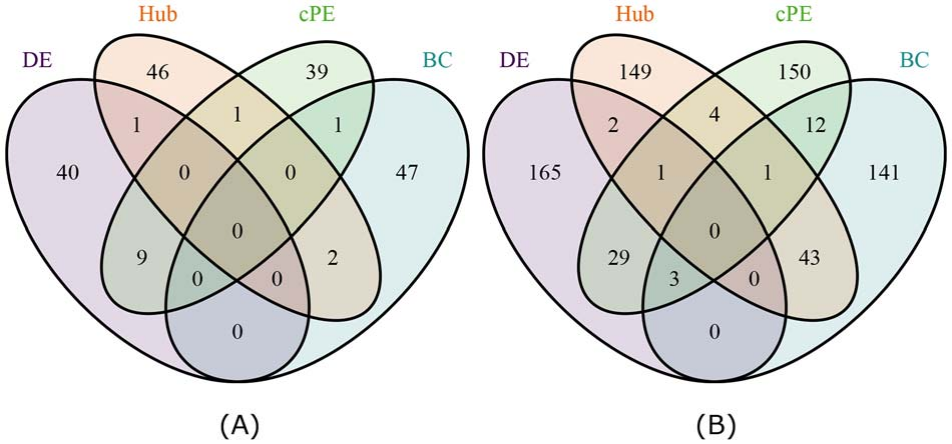
The overlap among differential expression (DE), betweenness centrality (BC), hub analysis (Hub) and the proposed method cPE. **(A)**. The overlap among four methods in their top 50 predicted PE-associated genes. **(B)**. Top 200 predicted PE-associated genes.

To determine if the four methods detect similar PE-associated genes, their identified genes in the top 50 and 200 predicted PE-associated genes were compared. Interestingly we found that the four methods have little overlap with each other (Figure 8). In the case of the top 50 PE-associated genes identified, cPE has nine, one and one genes which are also listed as DEGs, BC and hub genes, respectively. cPE has more overlapping genes with DE than BC and Hub, which is also seen in the cases of the top 200 identified PE-associated genes. This is expected as both DE and cPE rely on LogFC to rank the candidate PE-associated genes. We observed that the similarity between methods increased along with the ranked list of cPE (S1 Figure). Interestingly, cPE has more genes overlapped with BC and Hub than with DE after the top 441 and 524 genes, respectively. In general, cPE identified a greater number of genes which were not in the lists provided by other methods. Specifically, 85.2%, 64.1% and 52.6% cPE genes were not in the top 1722 DE, BC and Hub genes, respectively.

Some PE-associated genes have a known relationship with a gene mutation. For example, functional gene variations can affect the thrombogenic and angiogenic properties which can lead to abnormalities of the placenta and PE [54, 55]. Mutations in the genome can be single-nucleotide variants, insertions and deletions, copy number aberrations or structural variants. We used the PESNPdb database (last updated 26 September 2012) [56] of known SNPs with a link to PE (which provides different yet complementary information from dbPEC) to investigate how many SNPs were related to the genes identified by cPE. These PE-related SNPs may offer insight into the mechanisms of PE development. A total of 26 type-1 critical nodes and 12 type-2 critical nodes were in the list of PESNPdb (Table 2), which indicates these critical nodes might contribute to the genetic susceptibility of PE. In summary, 38 out of 1722 (2.2%) genes displayed PE-related SNPs in cPE, compared with 1.3% (52/4096) in DE, 1.9% (45/2317) in BC and 1.9% (83/4370) in Hub.

**Table 2:** Overlap between critical nodes and known PE-related SNPs as per the PESNPdb database

Node type	Genes
Type-1	AGTR1, APOB, CNR1, COMT, CX3CR1, DRD4, ENG, ESR2, ESRRG, GP1BA, HHEX, IFNG, IGF2R, IL12RB1, IL13, IL3, INHBB, LEP, LEPR, LNPEP, MMP9, NR1H2, PTGER2, SERPINE1, SHMT1, THBD
Type-2	ACVR1, ACVR2A, CCR5, COL1A1, CXCR4, F2, HBEGF, ICAM1, IGF1, IL4R, LPL, MMP3

Given the significant need for PE biomarkers with a high positive predictive value, the identified genes from control theory could also hold promise in biomarker research. Type-1 critical nodes included genes such as Leptin (LEP) [57], Endoglin (ENG) [58], Selectin P (SELP) [59], Pentraxin 3 (PTX3) [59], Angiotensin II Receptor Type 1 (AGTR1) [60], Podocalyxin Like (PODXL) [61] and Insulin Like Growth Factor Binding Protein 1 (IGFBP1) [62] which have well-known associations with PE and utility as biomarkers. Type-2 critical nodes included genes such as Caspase 3 (CASP3) [63], Heparan Sulfate Proteoglycan 2 (HSPG2) [64], Intercellular Adhesion Molecule 1 (ICAM1) [65], interleukin 8 (IL8) [66], Prostaglandin D2 Synthase (PTGDS) [67] and Vascular Cell Adhesion Molecule 1 (VCAM1) [68] that have associations with PE.

### 3.3 Understanding functions of the predicted novel PE-associated genes

Besides the validated PE-related genes in dbPEC, cPE also predicted novel PE-associated genes, which require further investigation. cPE can be used to shortlist genes of potential clinical relevance for PE to help direct wet-lab experiments. For example, 16 out of the top 20 cPE genes are present within dbPEC. There are four not listed, which are Olfactomedin Like 3 (OLFML3), Phosphatidylinositol-4, 5-Bisphosphate 3-Kinase Catalytic Subunit Beta (PIK3CB), Opioid Receptor Kappa 1 (OPRK1) and Proteoglycan 2 (PRG2). Previous studies have demonstrated these genes are differentially expressed in the preeclamptic placenta [69, 70]. OLFML3 is involved in embryonic development including the central nervous system, muscle development [71] and proangiogenic functions [72]. PIK3CB has been identified in several network studies, including for EOPE [73] and could help discriminate five distinct subtypes in PE [33] likely due to PIK3CB’s involvement in trophoblast differentiation [74]. OPRK1 also has been found to be differentially expressed in EOPE placentas [75]. The global RNA profiling of trophoblast subpopulations in severe PE placentas found that PRG2 was the most highly upregulated mRNA in syncytiotrophoblasts of severe PE placentas [70]. There is speculation that PRG2 may have clinical utility as a biomarker of severe PE [70]. Unfortunately, current research on the relationship between these four genes and PE is still limited.

cPE identifies many potential biologically meaningful genes in the top 50 predicted PE-associated gene list (S2 File). Within this list, 34 genes are present within dbPEC (2781 gene list), four genes translate four protein markers (LEP, ENG, IL8 and VCAM1) for PE and four genes (LEP, ENG, IGF1 and SERPINE1) contain known PE-related SNPs. Of the 16 genes not present in dbPEC most do have a known association with at least one form of PE, some of these proteins of interest include Solute Carrier Family 1 Member 6 (SLC1A6), Stanniocalcin-2 (STC2) and Gamma-aminobutyric acid receptor subunit beta-1 (GABRB1). Amino acid transporters such as SLC1A6 are vital for fetal growth with their expression likely being controlled by methylation which is altered in conditions such as gestational diabetes or PE [76]. STC2 involved in calcium and phosphate homeostasis, an inhibitor of PAPP-A known to affect IGF signaling [77] and has implications for pregnancy complications such as PE [78]. GABRB1 has previously been linked with preterm birth, its role in PE requires further investigation [79].

There were 1261 proposed PE-associated genes identified by cPE (from 1293 genes not listed in dbPEC) and 2397 PE-associated genes listed in dbPEC (from 2781 gene list) overlapping with the background genes (org.Hs.eg.db). These genes were used for enrichment analysis. The identified genes not listed in dbPEC (defined as proposed PE-associated genes) are significantly enriched (adjusted *P*-value less than 0.01) in 384 nonredundant GO terms of biological process subontology. Indeed, 172 out of the 384 GO terms identified for the proposed novel PE-associated genes are also in the 547 GO terms of PE-associated genes listed in dbPEC. This indicates that some proposed PE-associated genes are involved in the same biological processes or have similar functions to the confirmed PE-associated genes. There are 144 GO terms of proposed PE-associated genes which showed low similarity (less than 0.7) with the GO terms of the confirmed PE-associated genes. When ranking GO terms generated from the cPE list (decreasing adjusted *P*-value), the top two terms are positive regulation of kinase activity (GO:0033674) and positive regulation of MAPK cascade (GO:0043410). These terms are also in the top three when considering cPE genes which do not occur in dbPEC. Kinases and MAPK specifically play a role in cell survival, proliferation, metabolism and are associated with PE via regulation of trophoblast function/placentation [80]. Terms identified in cPE but not in dbPEC include sterol import (GO:0035376) and cholesterol import (GO:0070508) which are known to be critical in pregnancy maintenance [81] and SMAD protein complex assembly (GO:0007183) known to be associated with trophoblast invasiveness [82]. The full lists of GO terms can be found in S3 File.

The genes in dbPEC and proposed PE-associated genes not listed in dbPEC are significantly enriched (adjusted *P*-value less than 0.01) in 125 and 104 KEGG pathways, respectively (S4 File). There are 70 KEGG pathways which are overlapping between these two lists. Therefore, the proposed PE-associated genes likely participate in many of the same pathways implicated in the development of PE. Some KEGG pathways which are enriched in cPE but not found in dbPEC include Notch signaling pathway (hsa04330; mediates hypoxia-induced trophoblast migration), p53 signaling pathway (hsa04115; mediates apoptosis, altered in PE) and VEGF signaling pathway (hsa04370; altered in PE), all of which are known to be important in PE [83].

### 3.4 Identification of critical genes for two major subtypes of PE

PE is broadly separated into two subtypes: EOPE, which is defined as PE occurring before the 34th week of pregnancy and LOPE, defined as PE occurring after the 34th week of pregnancy. Significantly, although these subtypes are similar in their downstream clinical presentation (separated by time of symptom onset) they are thought to arise from different triggers at distinct gestational timings, in both cases leading to placental and vascular dysfunction. Specifically, EOPE is characterized by poor placentation (impaired extravillous trophoblast invasion) and spiral artery remodeling [6]. On the other hand, LOPE, the most common type of PE, arises after normal placentation through maternal factors including inflammation [6].

In this section, we investigated the critical genes for these PE subtypes. As discussed previously, PE is a heterogeneous disease with two major subtypes: EOPE and LOPE. As EOPE and LOPE have different clinical features, hemodynamic states and risk factors, the subtypes likely have different causes and genetic susceptibility. Although not considered in this work it is also important to note that within these subtypes disease severity can vary widely. For example, PE may or may not be associated with FGR and its presence or absence is an important consideration for data interpretation as it indicates the placenta’s inability to properly support fetal development. Indeed 36.2% of all PE cases are also associated with FGR (most of which occur in EOPE) [84]. As with the original study which collated these datasets [33], we assumed that biases (ethnicity, gestation, fetal gender) did not have a significant impact.

In the experiment, PE samples were divided into EOPE and LOPE samples based on gestation, irrespective of other clinical factors such as the presence of IUGR or other conditions. We applied cPE to the EOPE and LOPE gene expression data to identify critical genes for each subtype of PE and the genes which are critical in both subtypes. The DE analysis was then performed on the gene expression data of EOPE (or LOPE) and control conditions. LogFC and adjusted *P*-value were used to rank and select the candidate genes.

We identified 1057 type-1 and 716 type-2 critical nodes for EOPE, and 1032 type-1 and 683 type-2 critical nodes for LOPE. There are 494 type-1 and 298 type-2 critical genes that are specific to EOPE, and 469 type-1 and 265 type-2 critical genes for LOPE. 563 type-1 and 418 type-2 genes are critical for both subtypes of PE. The predicted subtype-specific genes and the genes which are critical for both EOPE and LOPE are listed in S5 File.

The identified genes which are not listed in dbPEC and have little known associations are of considerable interest potentially providing novel insights. For example, Proteoglycan 2 (PRG2), Ephrin A1 (EFNA1) and Kruppel Like Factor 6 (KLF6) identified as EOPE, LOPE and combined (LOPE/EOPE), respectively. PRG2 is toxic to mammalian cells and it is upregulated at the mRNA level in severe PE samples [70]. There is evidence that EFNA1 expressed exclusively in the invasive extravillous trophoblast cell lineage, suggesting that EFNA1 may participate in the targeting of the trophoblast to the uterine tissue and spiral arteries [85]. Its association with LOPE is interesting, considering LOPE is generally considered maternal in origin. KLF6 may mediate some of the effects of hypoxia in placenta development and so has relevance in the development of PE requiring further investigation [86]. Experimental validation is required for these genes identified with cPE to understand their potential roles in PE development.

It is important to note that only term samples were used as the control for EOPE, LOPE and PE samples. In general, experimental design considerations for preterm controls require thoughtful consideration due to difficulty in obtaining appropriate tissue. The ‘preterm controls’ within this dataset were mostly derived from placentas (<30 weeks) with signs of infection (predominantly chorioamnionitis) as discussed by Leavey *et al.* [33]. For this reason, these preterm controls were excluded from the subtype analysis with only term controls being used. When preterm controls were included in the analysis (data not shown, available at https://github.com/XiaomeiLi1/cPE) only the gene ranking was affected, with the critical genes identified remaining unchanged.

## 4 Discussion

The molecular mechanisms leading to PE development are still poorly understood. Identifying critical PE-associated genes will likely help elucidate their regulatory mechanisms and improve PE diagnosis and treatment. Increasing amounts of data generated from next-generation sequencing are providing great opportunities to uncover new insights into the molecular mechanisms of PE development. Computational methods have been developed for such a task, including differential expression, betweenness centrality and hub analysis. However, more effective approaches for detecting novel PE-associated genes are required.

We proposed a novel and effective control theory-based method to uncover critical PE-associated genes based on a directed PE-specific gene regulatory network. The PE-specific gene regulatory network was constructed based on placenta gene expression data and prior knowledge, such as TF genes and PPI interactions. Critical PE-associated genes were selected as they play important roles in the gene regulatory network of PE.

We have applied the proposed cPE method to one of the largest human placenta gene expression datasets for PE currently available. The cPE approach identified 1722 genes that are likely PE related, 24.9% of which are known to be associated with PE in the dbPEC database. Compared with standard methods, cPE recovered a higher proportion of validated PE-associated genes listed in dbPEC. More importantly, cPE has little overlap with genes identified with standard methods in the top 50 and 200 predicted PE-associated genes. There were 619 cPE genes that cannot be identified by any of the three methods, i.e. DE, BC and Hub. Therefore, cPE could provide a novel insight for PE-associated gene identification and could complement other existing methods. The results from all four methods could offer complimentary insights.

cPE can also be used to explore critical PE-associated genes for PE diagnosis or other related pregnancy complications. We demonstrated that cPE identified known PE-associated genes, genes that translate known protein markers for PE and genes that contain known PE-related SNPs. Moreover, cPE has identified genes that previously had little known association with PE; further work will be required to confirm these associations.

These results are dependent on the reliability of the microarray data and the DirectedPPI network. In the PE-specific network construction, the assumption has been implicit that the gene regulatory relationships are linear. Our network construction method, or any such method, cannot prove a non-association between genes. Therefore, we cannot conclude that the links not supported by the PCC test and the DirectedPPI network are not active gene relationships in PE. However, there is no statistical evidence for these links activating in the PE-specific network for the given microarray data and the DirectedPPI network. It is important to note, as with any network analysis, there might be missing or spurious links presenting in the network because of the quality of microarray data or directed PPI. However, the control theory method is robust to identify critical nodes in a PPI network. For example, it has been reported that 90% of critical nodes could be recovered by the control theory method when adding or removing links in the original network [28]. Therefore, the control-theory-based method likely identifies the most critical genes in the current PE-specific network.

There are multiple suggestions for future work. Firstly, while cPE does not require a control population to identify critical genes, a control population is, however, required for gene prioritization, as per differential expression analysis. In this instance, there are questions surrounding the prioritized critical genes identified for PE and the use of controls. Larger datasets (including single-cell sequencing) and more appropriate controls will undoubtedly demonstrate the true utility of the proposed approach. For example, Gong *et al.* recently published high depth sequencing for more than 300 placentas (including control, PE and FGR) from well-characterized placental phenotypes [87], which will undoubtedly be a valuable resource. In addition, we suggest incorporating additional clinical subtypes and sample types, particularly maternal blood samples throughout gestation (potentially animal and cellular models) into the cPE method. Based on these data, the discovery of critical PE-associated genes could hold great promise for identifying early diagnostic markers with high positive predictive value.

Secondly, within this study for simplicity PE samples were divided into EOPE and LOPE based on gestation. However, Leavey *et al.* who originally utilized the GSE75010 dataset used unsupervised clustering to further divide the data into five categories [33]. The utilization of cPE with further subtype delineation could provide even greater directed insights. Finally, experimental validation and systematic review of the identified PE-associated genes is warranted, especially for genes that have previously had little known association.

## 5 Conclusion

This work describes a computational approach that has identified PE-associated genes including ones that have previously had little known association with PE. Ultimately, this approach could eventually aid in a further understanding of PE molecular mechanisms, uncover biomarkers and contribute to improved PE diagnosis and treatment.

**Key Points**

- Preeclampsia is a pregnancy complication which can cause serious short- and long-term complications for both the mothers and their offspring.
- The precise molecular mechanisms which lead to the heterogeneous condition are poorly understood which has hampered development of new treatments, biomarker discovery and early diagnosis.
- We describe for the first time a control theory method to identify genes potentially associated with preeclampsia.
- Further expanding the proposed control theory approach with additional datasets and condition subtypes could improve the understanding of preeclampsia molecular mechanisms, aid in biomarker identification and ultimately contribute to improved preeclampsia diagnosis and treatment.

## Authors’ contributions

X.L. collected data, implemented the computational methods and performed the analysis. T.L. and M.W. jointly conceived and designed this work. X.L. and M.W. wrote the original draft. X.L., T.L., C.W., L.L., J.L, B.T. and M.W. interpreted the results. L.L., C.W., J.L. and B.T. revised the manuscript based on their respective expertise.

## Supplementary Material

S1_Figure_elac006

S1_File_elac006

S2_File_elac006

S3_File_elac006

S4_File_elac006

S5_File_elac006

## Data Availability

GSE75010 is available at the Gene Expression Omnibus database (https://www.ncbi.nlm.nih.gov/geo/). All other data are contained within the Article and its Supplementary files or available on https://github.com/XiaomeiLi1/cPE. The R scripts for this study are available on https://github.com/XiaomeiLi1/cPE.

## Footnotes

1
https://www.ncbi.nlm.nih.gov/geo/

2
https://www.flyrnai.org/DirectedPPI/Download.jsp

3
http://bejerano.stanford.edu/pesnpdb

## Author Biographies

**Xiaomei Li** is a PhD student at the University of South Australia. Her research interests are causal inference and its applications in Bioinformatics.

**Lin Liu** is an associate professor at UniSA STEM, University of South Australia (UniSA). She received her PhD degree in computer systems engineering from UniSA in 2006. Her current research interests include data mining/machine learning, causal inference and Bioinformatics.

**Clare Whitehead** is an obstetrician the Royal Women's Hospital (Melbourne) and senior research fellow at the University of Melbourne, Department of Obstetrics and Gynaecology. Her research interest includes the establishment of protein and genetic markers to monitor placental function.

**Jiuyong Li** is a professor at the UniSA STEM, University of South Australia. He received his PhD degree in computer science from Griffith University, Australia (2002). His research interests are in the fields of data mining, privacy preserving, causal discovery and Bioinformatics. His research has been supported by seven prestigious Australian Research Council Discovery grants.

**Benjamin Thierry** is the University of South Australia Professor of Bioengineering and a NHMRC Investigator Fellow. His research interests include biodiagnostics and nanomedicine in cancer and pregnancy.

**Thuc D. Le** is an associate professor at UniSA STEM, University of South Australia. He is an ARC DECRA fellow (2020–2022) and was an NHMRC ECR fellow (2017–2019). His research interests include causal discovery methods and their applications in Bioinformatics.

**Marnie Winter** is a Biomedical Engineer and research fellow at the Future Industries Institute, University of South Australia. Her research interests include the development of novel approaches to screen for pregnancy complications, including preeclampsia.
