# Supplementary figures and images for: Identifying preeclampsia-associated genes using a control theory method
Source: Brief Funct Genomics. 2022 Apr 28;21(4):296–309. doi: 10.1093/bfgp/elac006 (PMC9328024; doi:10.1093/bfgp/elac006)

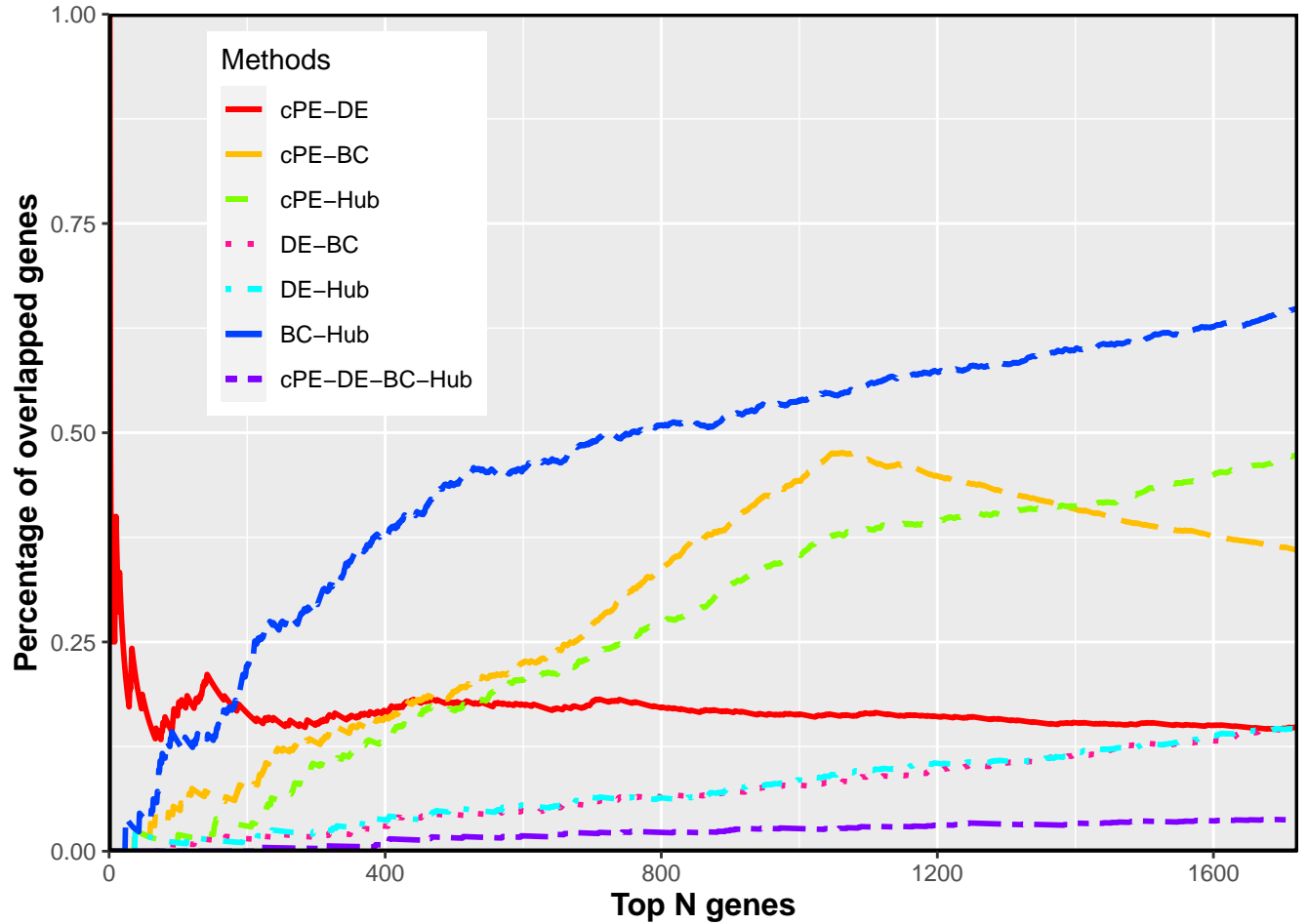

Supplement: S1_Figure_elac006 [file s1_figure_elac006.pdf]
